# Treatment Approaches to Molar Incisor Hypomineralization: A Systematic Review

**DOI:** 10.3390/jcm12227194

**Published:** 2023-11-20

**Authors:** Angelo Michele Inchingolo, Alessio Danilo Inchingolo, Fabio Viapiano, Anna Maria Ciocia, Irene Ferrara, Anna Netti, Gianna Dipalma, Andrea Palermo, Francesco Inchingolo

**Affiliations:** 1 Department of Interdisciplinary Medicine, School of Medicine, University of Bari “Aldo Moro”, 70124 Bari, Italy; angeloinchingolo@gmail.com (A.M.I.); ad.inchingolo@libero.it (A.D.I.); viapianofabio96@gmail.com (F.V.); anna.ciocia1@gmail.com (A.M.C.); ire.ferra3@gmail.com (I.F.); annanetti@inwind.it (A.N.); 2 College of Medicine and Dentistry, Birmingham B4 6BN, UK; andrea.palermo2004@libero.it

**Keywords:** molar incisor hypomineralization, enamel hypomineralization, treatment, enamel defect, therapy, children, sealant, pediatric dentistry, caseine, fluoride, preventive therapies, restorative therapies

## Abstract

Aim: This systematic review aimed to comprehensively evaluate the available literature on treating molar incisor hypomineralization (MIH) or enamel hypomineralization published between 2013 and 2023, focusing on identifying relevant studies and their characteristics. Materials and Methods: The search process encompassed reputable academic databases, including PubMed, Scopus, Cochrane Library, and Web of Science, using a precise keyword strategy (“((molar incisor hypomineralization) OR (enamel hypomineralization)) AND (treatment)”). A total of 637 articles were initially retrieved, followed by a strict selection process adhering to PRISMA guidelines. The inclusion criteria encompassed Randomized Control Trials (RCTs), case series with more than five clinical cases (CSs), studies involving human participants, availability as free full-text or accessible with university credentials, and English-language publications. Exclusion criteria included systematic or literature reviews, editorials, single-case reports, studies conducted in vitro, those involving animals, paid articles, and non-English-language publications. Results: The search yielded 864 articles, of which 23 met the stringent inclusion criteria after a meticulous selection process. These studies will serve as the basis for a comprehensive analysis of MIH treatment approaches. The systematic review ensures the quality and relevance of the chosen studies for a detailed assessment of MIH treatment strategies. Conclusions: This systematic review will provide valuable insights into the characteristics of selected studies, patient profiles, and available treatment options for molar incisor hypomineralization, contributing to a better understanding of this dental condition’s management.

## 1. Introduction

Molar incisor hypomineralization (MIH) constitutes an oral health challenge affecting numerous individuals globally. It is a developmental disorder of the dental enamel structure. The term MIH was first introduced in 2001 to describe a systemic condition that affects from one to four first permanent molars (FPM), frequently associated with affected incisors [1,2,3,4,5].

The etiology of MIH is intricate and not entirely elucidated, though it is believed to be multifactorial. Some studies propose that adverse events during prenatal development or early years of life, such as infections, febrile episodes, neonatal traumas, and specific childhood illnesses, may be linked to the onset of MIH. Exposure to environmental toxins, medication usage during pregnancy or childhood, and complications during childbirth have also been correlated with the condition. However, there is not a single identifiable causative factor for MIH, and further research is warranted to better grasp the underlying etiological mechanisms [6,7,8,9,10,11].

The MIH is an oral condition manifested through various dental enamel anomalies, predominantly in the first permanent molars, but it can also affect the permanent incisors. Clinically, the affected teeth may exhibit color variations, ranging from opaque white to yellow or brown. These opacities might be white, creamy, yellow, or brown in hue. One important finding is that post-eruptive breakdown (PEB) is more likely to occur the darker the opacity. Additionally, molars exhibit PEBs more frequently than incisors, most likely as a result of the larger masticatory pressures present in the molar region [12,13,14,15]. Additionally, the enamel’s integrity can be compromised, leading to fragility and being prone to fractures. Patients might also experience heightened dental sensitivity, especially when exposed to thermal or sugary stimuli. This condition not only impacts the teeth’s functionality, but can also have psychological ramifications, adversely influencing the patients’ self-esteem [16,17,18,19,20].

Several diagnostic techniques, including the developmental enamel defects (DED) and modified DDE index, the FDI, the modified Clarkson and O’Mullane DDE index, and the enamel defects index (EDI), have been proposed to evaluate and identify these developmental enamel defects (DDE). The modified DDE index is the most popular criterion for MIH categorization and the criteria recommended by the European Association of Paediatric Dentistry (EAPD) in 2003 [2,12,21,22,23].

**Figure 1:**
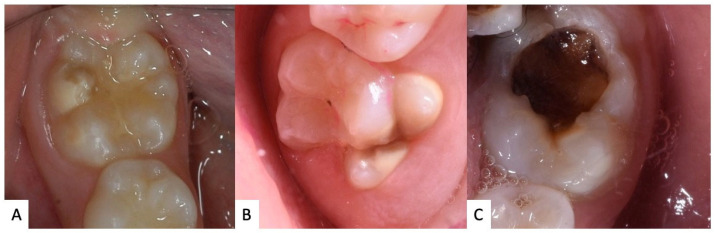
Examples of clinical manifestation of MIH: (**A**) mild, (**B**) moderate, (**C**) severe.

MIH can be classified based on the extent and severity of the lesions. There are primarily three degrees of severity: mild, moderate, and severe (Figure 1). In the mild form, there are typically opaque white enamel discolorations (Figure 1A). The moderate degree manifests with more pronounced discolorations, which can range from yellow to brown (Figure 1B). In the severe form, in addition to discolorations, there is a noticeable loss of hard tissue, leading to the formation of cavities or enamel fractures [8,24,25,26,27,28] (Figure 1C).

The diagnostic criteria established by Weerheijm et al. [2,29,30,31,32] have become foundational in recognizing and classifying MIH. According to these criteria, the diagnosis of MIH is primarily based on the clinical presentation of demarcated opacities in the enamel of affected teeth. These opacities can range in color from white to yellow or brown. Post-eruptive enamel breakdown, often resulting from the fragile hypomineralized enamel, is another characteristic feature. In more severe cases, MIH might lead to atypical restorations (specifically in molars), or even the extraction of affected molars due to extensive structural compromise [2,33,34,35,36,37,38,39].

The International Caries Detection and Assessment System (ICDAS) criteria have been established to provide a comprehensive and nuanced framework for the identification and classification of dental caries, from initial enamel changes to extensive cavitation [40,41,42,43,44,45]. When considering MIH, the ICDAS offers a consistent approach for evaluating the extent and severity of enamel defects. MIH manifests as distinct opacities in the enamel, ranging from white to yellow-brown discolorations. Utilizing the ICDAS criteria, clinicians can classify these enamel anomalies in a standardized manner, aiding in the distinction between early enamel changes and more advanced structural anomalies:

1. **ICDAS I**: This grade refers to the earliest visible changes in enamel translucency after prolonged air drying. For MIH, it can manifest as slight changes in enamel opacity, indicating hypomineralization without any structural loss.
2. **ICDAS II**: At this stage, distinct visual changes in enamel are evident even without air drying. The enamel might show white or yellowish opacities indicating a more pronounced hypomineralization, but there is still no cavitation or loss of enamel structure.
3. **ICDAS III**: This level denotes localized enamel breakdown due to MIH. While there is no cavitation into the dentin, the enamel may have micro-cavities or signs of minor structural loss due to the weakened hypomineralized structure [21,42,46,47,48,49].

**Figure 2:**
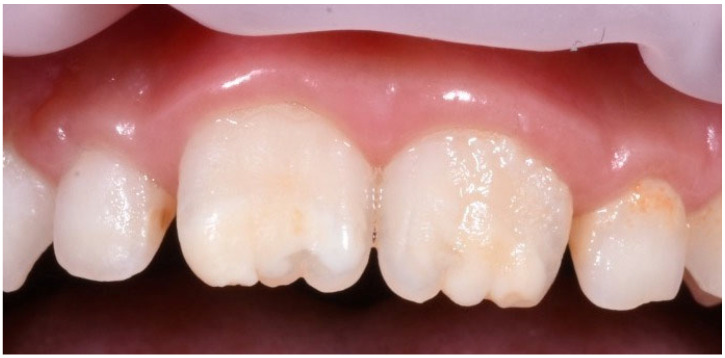
Examples of clinical manifestation of incisor affected by MIH.

The diagnosis of MIH must be made with caution, differentiating it from other conditions that may present with similar enamel anomalies. Among these, dental fluorosis, caused by excessive fluoride intake during tooth formation, can display streaks or opaque white spots on the enamel. Other pathologies, such as amelogenesis imperfecta, a genetic condition, can result in underdeveloped or absent enamel. The key distinction from MIH is that amelogenesis imperfecta affects all teeth, whereas MIH selectively targets the first molars and incisors (Figure 2). An accurate diagnosis is essential for appropriate and timely treatment [25,41,50,51,52,53,54].

In patients with MIH, prevention plays a pivotal role in averting further complications and ensuring oral health. The foremost preventive measure is the regular use of fluoride toothpastes and rinses with fluoridated solutions, as fluoride can aid in strengthening and remineralizing compromised enamel areas. It is also vital to attend regular dental check-ups, which allow early diagnosis and prompt intervention in the event of new lesions or caries [2,55,56,57].

Patients with MIH often exhibit teeth with heightened sensitivity and, at times, compromised enamel that might be more susceptible to mechanical and chemical damages. Therefore, it is imperative to adopt dietary habits that safeguard and maintain dental health. Firstly, patients should curtail the consumption of acidic foods and beverages (such as carbonated drinks, fruit juices, and fermented foods) that might further erode the enamel. Avoiding overly hard or crunchy foods, like nuts, candies, and ice, can assist in preventing the fracture of compromised enamel. Consuming calcium-rich foods, like milk, yoghurt, and cheese, can bolster dental health. Lastly, limiting sugary foods and drinks diminishes the risk of caries, a further threat to teeth with MIH [58,59,60,61,62,63].

The treatment of MIH is often challenging due to the variability of clinical manifestations and the potential sensitivity of the affected teeth. The primary aim of treatment is to reduce dental sensitivity, protect compromised enamel, and enhance aesthetics. In mild cases, desensitizing agents and fluoride can be used to alleviate sensitivity and prevent caries. For moderate cases, adhesive fillings and sealants might be applied to shield the enamel and provide a functional chewing surface. In instances where MIH is severe, restorative techniques such as dental crowns may be necessary. The therapeutic approach should be individualized based on the severity of MIH, patient needs, and available treatment options. Prevention and early diagnosis are paramount to ensuring effective treatment and minimizing complications associated with MIH [1,41,64,65,66].

MIH has garnered increasing attention in recent years due to its widespread presence across various populations. The prevalence of MIH displays a broad range of variations depending on the regions and populations studied. Global studies have reported a prevalence ranging from 2.4% to 40% in children between the ages of 7 and 13. This wide range can be attributed to various factors, such as differences in diagnostic criteria, study methodologies, and environmental and genetic factors. It is important to underscore that, despite regional variations, MIH has been identified as a significant clinical issue in many nations, emphasizing the need for a deeper understanding and effective preventive strategies [25,67,68,69].

This article aims to conduct a systematic review of the available treatments for MIH, to provide a clear and up-to-date overview based on the latest scientific evidence. This review seeks to serve as a guide for clinicians in selecting the most appropriate therapeutic approach, considering both treatment efficacy and the patient’s experience.

## 2. Materials and Methods

**Figure 3:**
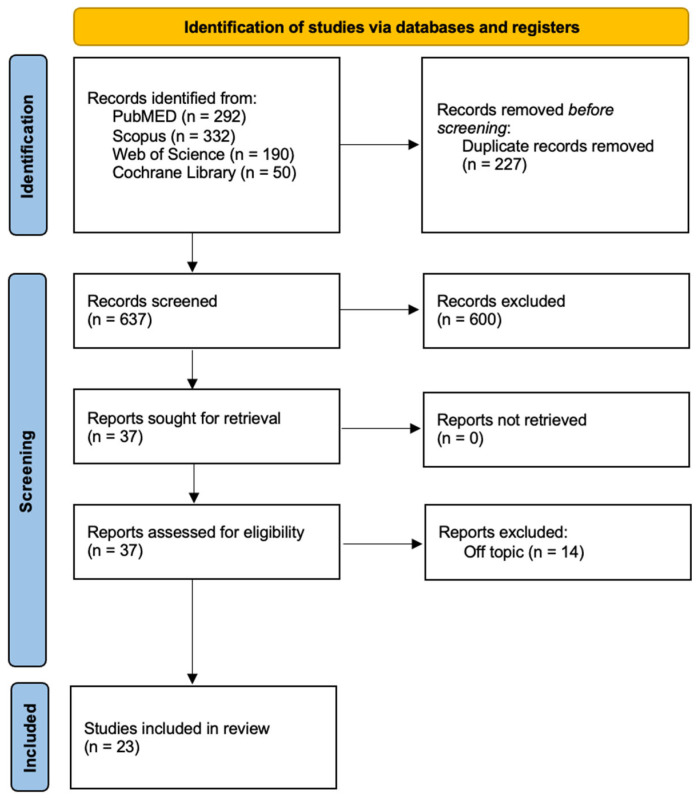
PRISMA flowchart diagram of the inclusion process.

The systematic review’s search process was conducted across multiple databases, including PubMed, Scopus, Cochrane Library, and Web of Science, to comprehensively gather studies on the treatment of molar incisor hypomineralization (MIH) or enamel hypomineralization published between 2013 and 2023. The search strategy employed the following keywords: “((molar incisor hypomineralization) OR (enamel hypomineralization)) AND (treatment)”. This search strategy yielded a total of 637 articles within the specified timeframe. Subsequently, the articles underwent a systematic selection process adhering to PRISMA guidelines, leading to the identification of 23 articles that met the inclusion criteria and were deemed appropriate for inclusion in the systematic review (Figure 3). The search and selection of articles for this systematic review were conducted following inclusion and exclusion criteria. Inclusion criteria comprised articles that met the following conditions: (1) Randomized Control Trial (RCT) or randomized controlled clinical trial (RCCT), (2) case series with more than 5 clinical cases (CS), (3) studies involving human participants, (4) availability as free full-text or accessible with university credentials, and (5) articles published in the English language. Conversely, exclusion criteria were defined as follows: (1) systematic or literature reviews, (2) editorials, (3) case reports (those with only 1 case), (4) case series with fewer than 5 cases, (5) articles conducted in vitro, (6) studies involving animals, and (7) articles not published in the English language. These criteria were applied during the article selection process to ensure the quality and relevance of the chosen studies for the systematic review. These 23 selected articles will form the foundation for a comprehensive analysis of the available literature on MIH treatment approaches (Table 1).

### Quality Assessment

The quality of the included papers was assessed by two reviewers, RF and EI, using the ROBINS, which is a tool developed to assess the risk of bias in the results of non-randomized studies that compare the health effects of two or more interventions. Seven points were evaluated and each was assigned a degree of bias. A third reviewer (FI) was consulted in the event of a disagreement until an agreement was reached.

## 3. Results

The systematic review commenced with an extensive search across four major databases: Pubmed (292 articles), Scopus (332 articles), Cochrane Library (50 articles), and Web of Science (190 articles), resulting in the identification of 864 articles. After the removal of duplicates (227 articles), a total of 637 studies remained. Subsequently, a screening process involving the analysis of article titles and abstracts led to the exclusion of 601 articles that did not meet the predefined inclusion criteria. Following this selection, the authors further assessed the eligibility of the remaining 37 articles and excluded 14 publications, as they were found to be unrelated to the review’s focus. Ultimately, the systematic review included a final set of 23 studies for qualitative analysis, ensuring that only the most relevant research was incorporated into the review’s comprehensive assessment. The articles included in our review are summarized in Table 1.

### Quality Assessment and Risk of Bias

**Figure 4:**
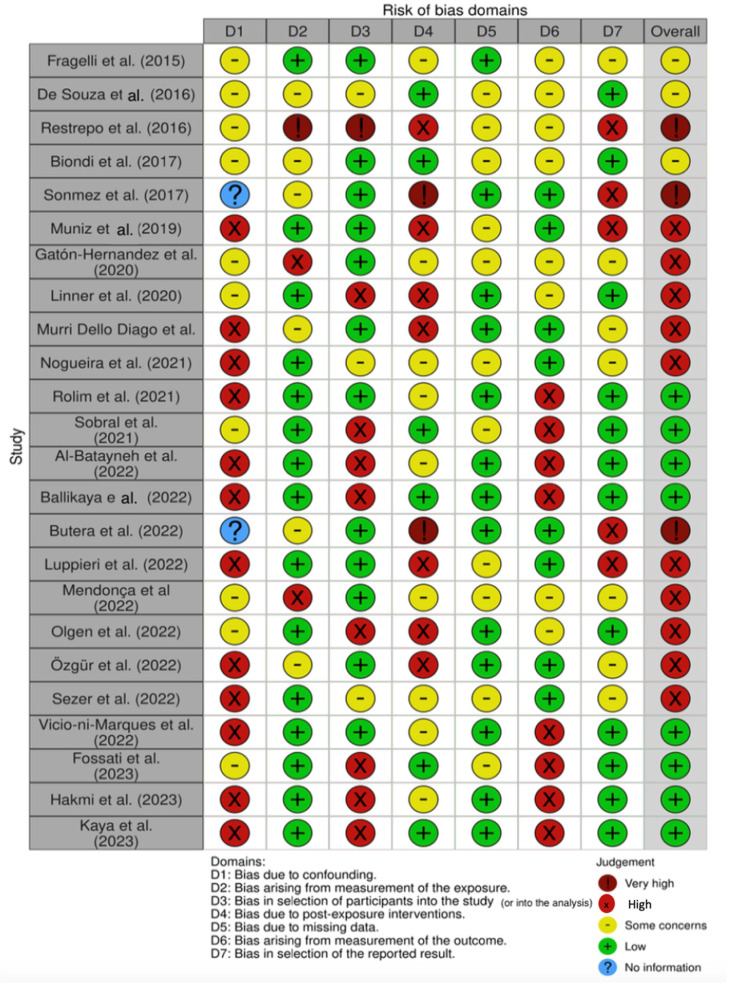
Risk of bias [3,57,64,70,71,72,73,74,75,76,77,78,79,80,81,82,83,84,85,86,87,88,89,90].

The risk of bias in the included studies is reported in Figure 4. Regarding the bias due to confounding, most studies have a high risk. The bias arising from measurement is a parameter with a low risk of bias. Many studies have a low risk of bias due to bias in the selection of participants. Bias due to post-exposure cannot be calculated due to high heterogeneity. The bias due to missing data is low in many studies. Bias arising from the measurement of the outcome is low. Bias in the selection of the reported results is high in most studies. The final results show that 10 studies have a high risk of bias, 3 have a very high risk of bias and 8 have a low risk of bias.

## 4. Discussion

### 4.1. Therapy for Dental Hypersensitivity and Pulpotomy

Dental hypersensitivity is a significant clinical problem associated with MIH, a condition that mainly affects the first permanent molars and sometimes the incisors. This condition is characterized by the hypomineralization of dental enamel, which makes it more susceptible to damage and painful sensations. MIH hypersensitivity can cause significant discomfort to patients, making it difficult to consume cold or hot food and drinks, as well as normal oral hygiene activities.

Alberto Murri Dello Diago and Patricia Gatón-Hernandez et al. [76] outlined the definition and prevalence of MIH, emphasizing its systemic nature and geographical variability. They describe in detail the clinical features of enamel hypomineralization in patients with MIH and its consequences, such as tooth sensitivity and reduced quality of life. The proposed treatment for MIH should be minimally invasive, aiming to protect, strengthen and preserve tooth structure, but there is currently a lack of specific guidelines for this condition [57,76].

The studies conducted by Ana Laura Fossati et al. and Ana Paula Taboada Sobral et al. [88] explored the role of photobiomodulation and glass ionomer sealant as complementary therapies for hypersensitivity in patients with MIH.

Both studies propose treatment protocols and clinical evaluations to determine the efficacy of these therapies, recognizing the importance of hypersensitivity management for the overall success of MIH treatment [79,88].

Fernanda L. Mendonça et al. conducted a randomized clinical trial comparing different therapies for MIH-associated tooth sensitivity in children. This study aims to develop a treatment protocol for tooth sensitivity in patients with MIH [84].

Ola B. Al-Batayneh et al. examined the success of different vital pulp therapy procedures in young permanent molars with MIH, confirming that pulp therapy can be an effective option to preserve tooth structure and provide symptom relief [80].

Fernanda Vicioni-Marques et al. addressed to the problem of hypersensitivity in patients with MIH and proposed the use of preventive analgesia as a method to reduce trans- and post-operative hypersensitivity during dental procedures. Their study demonstrates the effectiveness of preventive analgesia with ibuprofen in improving anesthesia and reducing hypersensitivity in children with MIH [87].

### 4.2. Dental Sealants and Application of Fluoride Paints and Casein Products

In the management of molar incisor hypomineralization (MIH), the application of fluoride varnishes (FVs) and casein-based products has emerged as a pivotal preventive and therapeutic strategy. Fluoride varnishes function by promoting remineralization of the affected enamel, thereby enhancing resistance to dental caries and reducing dentinal hypersensitivity. They form a temporary protective layer over the hypomineralized areas, slowing down enamel demineralization and enhancing the fluoride uptake by the tooth surface. On the other hand, casein phosphopeptide–amorphous calcium phosphate (CPP-ACP) based products, derived from casein, have shown promise in managing MIH [91]. CPP-ACP acts by maintaining a supersaturated environment of calcium and phosphate ions at the tooth surface, thus promoting remineralization and inhibiting further demineralization of the enamel. Together, the synergistic use of fluoride varnishes and casein-based products provides a holistic approach to halt the progression of MIH and restore the structural integrity of the affected teeth [77].

Based on the research conducted by Biondi et al. [72], the study’s main objective was to evaluate and compare the variations in mineral density observed in teeth with MIH following the application of varnishes that contain either 5% fluoride, 5% fluoride combined with tricalcium phosphate, or CPP-ACP. The results demonstrated significant differences in the percentage variation in fluorescence between the products used, especially in mild and moderate lesion groups. Among the products, Duraphat^®^ displayed the most effective results in improving mineral density for moderate lesions, with Clinpro^®^ being most effective in mild lesions [72].

Restrepo et al. [71] investigated the effects of fluoride varnish. The patients were randomly divided into two groups: one receiving four applications of 5% NaF varnish at one-week intervals and a control group undergoing usual home care. The primary assessment tool for the study was the quantitative light-induced fluorescence (QLF) method, which gauged changes in the fluorescence and size of lesions in anterior teeth treated with the varnish. The study revealed no significant changes in the fluorescence and area of MIH lesions over time after the application of the FV. This suggests that the architecture and protein/mineral content of the affected enamel might resist attempts at mineral incorporation. The conclusion was that while FV did not significantly remineralize MIH lesions, its application could still be beneficial for reducing the risk of dental caries and managing hypersensitivity associated with MIH [2]. In Nogueira et al.’s study [77], participants were randomly allocated to receive FV, fluoride varnish with phosphoric acid pre-treatment (FV + etch), and resin infiltration (RI). By the end of 18 months, the success rates of the interventions were measured, and the research identified various factors like DMFT (Decayed, Missing, and Filled Primary Teeth) index, opacity color, and age that were significantly associated with treatment failure; resin infiltration emerged as a more successful intervention compared to the other two methods, especially in participants aged 6–9 years with certain dental characteristics [77].

The study conducted by Sezer et al. [3] sought to assess the remineralization of demarcated hypomineralized opacities in incisors affected by MIH over three months, monitored using laser fluorescence (LF). Participants were randomized into three treatment groups: calcium glycerophosphate (CaGP), casein phosphopeptide amorphous calcium fluoride phosphate (CPP-ACFP), and a control group. The study employed specific dental care products for each group, with detailed instructions provided for daily oral hygiene routines. Compliance and adherence to instructions were regularly checked. The findings suggest the potential efficacy of both CaGP and CPP-ACFP in promoting remineralization in MIH-affected incisors. However, CPP-ACFP demonstrated the highest percentage change in lesions, with an LF score below 20, indicating its possible superior remineralizing properties in certain conditions. Importantly, no adverse effects or injuries were reported throughout the study, underscoring the safety of the interventions [3].

Olgen et al. [85] sought to understand the efficacy of various treatments for children diagnosed with MIH, focusing on first molars showing specific colorations indicative of MIH. The participants’ selection strictly adhered to the ICDAS II criteria and the MIH diagnosis criteria established by Weerheijm et al. [2] The children were randomly divided into four groups: the first group received 5% sodium FV applications, the second group was treated with a CPP-ACP paste, the third group used a CPP-ACFP paste, and the fourth group only received oral hygiene training. The evaluations were conducted at regular intervals over 24 months. The final outcomes showed that all remineralization agents used (FV, CPP-ACP, and CPP-ACFP) improved remineralization rates in both creamy-white- and yellow-brown-colored defects by the end of the study. Notably, FV took longer to show their effects compared to the pastes. The most promising results for the long-term preservation of teeth with yellow-brown defects were observed with pastes containing calcium and phosphate ions [85].

### 4.3. Direct and Indirect Resin and Composite Restorations

In mild and severe cases, molars with MIH often require restorative interventions to ensure long-term oral health and function. Numerous studies explore the treatment of MIH in such cases, focusing on composite restorations as a viable treatment approach [19]. By addressing the unique challenges presented by teeth with MIH and investigating the clinical performance of composite restorations, this study aims to provide valuable insights into improving the management of this complex dental condition in pediatric populations.

One study by Sonmez [73] focused on evaluating the resin-to-hypomineralized porous enamel micro shear binding strength, indicating that it is lower in MIH-affected molars compared to regular enamel. This often leads to secondary caries and degradation of enamel next to restorations, necessitating additional restoration and contributing to the loss of tooth structure [73,92,93].

Sonmez’s study involved a thorough evaluation of 70 children aged 8–12 with MIH, specifically assessing different cavity designs and deproteinization for the treatment of MIH-affected posterior molars (PFMs). The children were divided into various treatment groups: invasive and noninvasive treatments with differing cavity designs, along with a control group. The restorations for all groups involved etching with phosphoric acid, applying a self-etch adhesive, and using a nano-hybrid composite material.

The results of this study demonstrated that noninvasive treatment with a specific cavity design showed limitations in long-term management, with the control group (conventional resin cavities) exhibiting better overall restoration survival. Furthermore, the study indicated that the deproteinization of hypomineralized enamel significantly improved composite resin restoration (CRR) retention rates [73].

Another study by Kaya et al. [90] conducted a randomized clinical trial comparing two restorative materials, glass hybrid (GH) and direct composite with short-fiber-reinforced composite (SFRC) in terms of their clinical success and survival rates in molars affected by MIH. The findings revealed that SFRC outperformed GH restorations, particularly in terms of retention at 24 months [90].

Luppieri et al. [83] explored the use of resin infiltration (RI) as a treatment technique for MIH-affected permanent first molars (PFMs) in a clinical setting. The results highlighted a significant improvement in dentin hypersensitivity after resin infiltration, especially in severe MIH cases [83].

A study by Rolim et al. [78] conducted a randomized clinical trial comparing total-etch (TE) and self-etch (SE) adhesive protocols for the restoration of MIH-affected first permanent molars (FPMs). The findings indicated similar restoration survival rates for both protocols at 12 months, suggesting that both approaches can achieve comparable success while reducing pain and anxiety reported by the children [78].

Nogueira’s study compared the efficacy of fluoride varnish, fluoride varnish after enamel etching, and resin infiltration in preserving the structural integrity of MIH-affected teeth. Resin infiltration demonstrated a significantly lower rate of post-eruptive enamel breakdown compared to fluoride varnish treatments [77].

In a study by Özgür et al. [86], giomer- and conventional resin-based sealants were compared in their clinical success for MIH-affected molars. The study rejected the null hypothesis, demonstrating that conventional resin-based sealants had superior clinical success compared to giomer sealants [86].

Frangelli et al. [64] assessed the clinical performance of glass ionomer restorations in MIH-affected molars. The study highlighted a favorable likelihood of restored teeth maintaining their integrity, emphasizing the importance of a less invasive approach until the child matures for more complex procedures [64].

Linner et al. [75] conducted a retrospective cohort study comparing different treatment strategies for MIH-affected teeth. The results emphasized the effectiveness of conventional composite restorations and CAD/CAM ceramic restorations in patients affected by MIH, showcasing significantly higher success rates compared to other treatment options. The study emphasized the importance of considering patient cooperation, defect characteristics, and treatment options when managing MIH-related dental issues. It highlighted the benefits of more invasive but durable treatment approaches in adequately cooperative individuals [75].

Hakmi’s study evaluated both direct and indirect resin composite restorations for MIH-affected molars, demonstrating that both approaches were effective. Indirect resin composite restorations were particularly advantageous in terms of child satisfaction due to shorter treatment sessions [89,94,95,96,97].

Lastly, de Souza JF et al. conducted a comprehensive clinical trial evaluating the performance of direct composite resin restorations with two different adhesive systems for molars affected by MIH. The study found that both self-etching and total-etch adhesives can be effectively used for restorations in MIH-affected molars, resulting in good clinical success rates. The researchers concluded that when the cavity preparation is conservative, both self-etching adhesives and total-etch adhesives can be used for restorations in molars affected by MIH, resulting in good clinical success rates. They also noted that modern adhesives may be a reasonable choice for such molars, but emphasized the importance of considering the bonding capability of cavosurface margins in hypomineralized enamel [70].

Overall, these studies contribute valuable insights into the clinical evaluation and treatment of MIH, highlighting the importance of choosing appropriate restoration techniques and materials to enhance the long-term outcomes and structural integrity of affected teeth. Further research and exploration in this area are warranted to refine treatment approaches and improve the management of MIH in pediatric populations.

### 4.4. Hydroxyapatite

Butera et al. [82] conducted a split-mouth randomized controlled clinical trial to evaluate the treatment of molar incisor hypomineralization (MIH) in pediatric patients. It involved pediatric patients with MIH. The intervention involved the use of a hydroxyapatite-based paste applied to MIH-affected teeth in one random quadrant (the test group), while the contralateral teeth did not receive the paste (control group). The study assessed various outcome measures, including plaque control, bleeding index, MIH Treatment Need Index (MIH-TNI), and Schiff Air Index (SAI) at multiple time points over a 9-month period. The results indicated that the hydroxyapatite-based paste had a desensitizing effect and led to a slight reduction in MIH-TNI scores in teeth with mild MIH defects. The study concluded that the paste could be recommended for domiciliary use in MIH patients to reduce dental sensitivity and improve enamel integrity. Further research and evaluation were suggested to explore the paste’s effectiveness in more severe MIH cases and to assess its long-term effects [82].

### 4.5. Low-Level Laser Therapy

Muniz et al. [74] carried out a randomized clinical trial involving 66 children with a total of 214 teeth affected by molar-incisor hypomineralization (MIH). The study took place at a pediatric dentistry clinic in São Luís, Brazil, between March and December 2018. The inclusion criteria required children to be between 6 and 12 years old and have at least one erupted permanent first molar or incisor with hypomineralization and sensitivity. The study employed various treatments, including low-level laser therapy (LLLT), fluoride varnish (FV), or a combination of both (L + FV), with sensitivity as the primary outcome measure. The findings indicated that the combination of LLLT and fluoride varnish had a similar desensitizing effect to fluoride varnish alone at the end of treatment. However, LLLT provided immediate relief, while fluoride varnish had a delayed effect. The authors also suggested that LLLT’s anti-inflammatory properties contributed to reducing sensitivity in teeth affected by MIH. This study is significant for pediatric dentists, as it offers clinical insights into the management of MIH, highlighting the effectiveness of LLLT as a treatment option for desensitizing affected teeth, especially when combined with fluoride therapy [74].

### 4.6. Fluorinated Silver Diamine Therapy

Ballikaya et al. [81] conducted a randomized, prospective study, in which researchers investigated the treatment of teeth affected by MIH (molar incisor hypomineralization) in children aged 6 to 13 years. The researchers employed a split-mouth design, blinding was not possible due to the procedures, and treatments were administered by experienced dentists. The study found that both silver diamine fluoride (SDF) application alone and SDF followed by Hybrid Glass Ionomer Cement (SMART) sealants were effective in treating MIH-affected molars, with reasonable retention rates and desensitization. Marginal discoloration due to SDF application was a common drawback in SMART sealants. This study suggests that these minimally invasive treatments are effective for MIH-affected molars, especially in children [81].

### 4.7. Biomimetic Mineralization

The available treatment modalities have primarily focused on mitigating the clinical consequences of MIH, such as restorations, sealants, and fluoride applications. However, recent breakthroughs in biomimetic mineralization have sparked new hope for addressing the underlying structural deficiencies in MIH-affected teeth, potentially revolutionizing the management of this condition. Biomimetic mineralization is a concept rooted in the imitation of nature’s own biomineralization processes, which creates and repair dental tissues [98].

It seeks to replicate the intricate molecular and structural dynamics involved in enamel and dentin formation. In the context of MIH, biomimetic mineralization offers a promising avenue for not only restoring the damaged enamel, but also promoting the regeneration of affected dental tissues. This approach, if successful, has the potential to go beyond conventional treatments that merely manage symptoms, enabling the restoration of the tooth’s natural integrity [99].

At the core of biomimetic mineralization in MIH treatment lies the emulation of enamel biomineralization, which is a finely orchestrated process involving ameloblasts, matrix proteins, and hydroxyapatite crystallization. By understanding the molecular and structural intricacies of enamel formation, researchers aim to engineer materials and techniques that can guide the growth of enamel-like structures on MIH-affected teeth. This endeavor includes the development of bioactive materials, scaffoldings, and signaling molecules that can facilitate the regeneration of enamel with improved strength and resistance to demineralization. In essence, biomimetic mineralization seeks to harness the body’s innate regenerative potential to repair MIH-affected teeth from within [100].

One promising approach within biomimetic mineralization is the utilization of enamel matrix proteins, such as amelogenin, to facilitate enamel-like tissue regeneration. These proteins play a pivotal role in enamel formation during tooth development. Incorporating enamel matrix proteins into MIH treatment may promote the proper alignment and growth of hydroxyapatite crystals, thereby enhancing the structural integrity of the enamel. Additionally, advanced techniques in tissue engineering, such as three-dimensional printing and nanotechnology, have enabled the precise control of material properties and structures, further facilitating biomimetic mineralization efforts [101].

Another intriguing aspect of biomimetic mineralization in MIH management is the potential for personalized treatment. Given the inherent variations in the severity and extent of MIH-affected teeth, tailoring treatment strategies to individual cases becomes increasingly important. Biomimetic mineralization, with its ability to mimic natural enamel formation, holds promise in the customization of treatment approaches. Researchers are exploring the use of patient-specific data, including genetic and clinical information, to design personalized biomimetic therapies that address the unique needs of each affected tooth [102].

While the concept of biomimetic mineralization in MIH treatment is undoubtedly promising, it is important to acknowledge the current challenges and limitations. Research in this field is still in its nascent stages, and many questions regarding the optimal biomaterials, techniques, and long-term outcomes remain unanswered. Clinical trials and rigorous testing are essential to validate the safety and efficacy of biomimetic mineralization approaches. Additionally, the cost-effectiveness and accessibility of such treatments must be considered, as advanced biomimetic therapies may be resource-intensive [103].

In conclusion, biomimetic mineralization represents an exciting frontier in the treatment of molar incisor hypomineralization (MIH). As research in this field continues to evolve, it is imperative to foster collaboration between dental practitioners, researchers, and material scientists to translate these promising concepts into effective, safe, and accessible treatments for MIH.

## 5. Limitations

- Limitation of the availability of long-term studies: Many of the studies included in the systematic review have a limited time duration (often 24 months or less). This limitation prevents long-term evaluation of the efficacy and stability of treatments for MIH, which may manifest different results over the years.
- Heterogeneity of treatment protocols: The studies included in the review use a variety of treatment protocols, including different product formulations and procedures. This heterogeneity may make it difficult to directly compare results between studies and determine the most effective treatment.
- Small sample of patients: Some studies involved a small number of participants, which may limit the ability to generalize results to a larger population of patients with MIH.
- Lack of standardization of assessment parameters: Studies have used a variety of assessment parameters, such as different caries indices or tooth sensitivity scales. The lack of standardization could complicate direct comparison of results between studies.
- Lack of long-term follow-up: some studies may not have provided long-term follow-up of treated patients, limiting understanding of the long-term effectiveness of treatments.

## 6. Conclusions

The evidence collected indicates that the treatment of MIH should be customized according to the severity of the case, the age of the patient, and other variables. There is no universal approach that is effective for all patients with MIH. Despite advances in the understanding and treatment of MIH, there are still many gaps in the scientific literature. It is essential to conduct further research to consolidate the available evidence, in particular long-term studies evaluating the efficacy and stability of treatments over the years. Based on the current evidence, it is possible to develop sound evidence-based clinical recommendations for the treatment of MIH. However, these recommendations should be periodically reviewed and updated according to new research findings. The management of MIH requires active patient and parental involvement. It is important to educate patients about their condition and encourage them to follow the treatment recommendations to achieve the best possible results. In conclusion, MIH is a complex condition that requires a personalized and evidence-based approach. Further research is needed to fill knowledge gaps and develop more robust clinical recommendations for the treatment of this condition.

## Figures and Tables

**Table 1:** Descriptive summary of item selection.

Author (Year)	Study Design	Number of Patients	Average Age	Gender	Severity of MIH	Treatment	Outcomes
Fragelli et al. (2015) [64]	RCT	21	7.7 years old (ranging from 6.37 to 9.54 years old)	12 males, 9 females	Mild, moderate, and Severe	Teeth affected by MIH were restored with glass ionomer cement (GIC). The treatment approach included conservative restoration without the complete removal of the MIH-affected area and control after 12 months	The chance of a repaired tooth remaining unaltered after a year was 78%, especially in single-surface tooth restorations. Invasive treatment should be postponed until the child is grown enough to cooperate with the therapy, which should be limited to teeth on just one surface
De Souza et al. (2016) [70]	RCT	26	6–8 years old	14 females, 12 males		A total of 41 PFMs were treated with direct composite resin restorations, which were randomized into two groups: self-etching adhesive (SEA) and total-etch adhesive (TEA). Control during 18 months	SEA and TEA both had acceptable clinical success rates in restoring molars affected by MIH, especially when the cavity preparation was conservative
Restrepo et al. (2016) [71]	RCT	51	9–12 years old	35 males and 17 females.	Mild, moderate, and severe	Study group: Four applications of 5% NaF varnish with one-week interval. Control Group: Usual home care-control. The fluoride varnish used was Duraphat^®^, Colgate Palmolive, Hamburg, Germany	The application of fluoride varnish on MIH lesions showed no significant changes in both mean levels of fluorescence and area of lesion over time. QLF method was used to monitor changes in fluorescence and extension of lesions. No favorable effect was observed on the remineralization of MIH lesions in anterior teeth after four applications of fluoride varnish
Biondi et al. (2017) [72]	RCT	55	6–17 years old	n.d.	Mild to moderate, with loss of structure	29 children (2013)—3 applications of 5% sodium fluoride varnish (Colgate Duraphat^®^) for 1 min 16 children (2014)—treatment with casein phosphopeptide-amorphous calcium phosphate CPP-ACP (MI Paste™ Recaldent^®^, GC America, Alsip, Illinois, USA) 10 children (2015)—5% sodium fluoride varnish containing tricalcium phosphate (TCP) (Clinpro™ White Varnish, 3M ESPE, St. Paul, MN, USA)	Mineral density of 92 teeth with MIH was assessed using DIAGNOdent. Percentage of variation in the three studied products was analyzed in each group. Duraphat^®^ showed the best results in moderate lesions, Clinpro^®^ in mild lesions
Sonmez et al. (2017) [73]	RCT	70 (126 teeth: 95 MIH PFMs; 31 caries but not MIH PFMs)	8.88 years old	n.d.	Moderate, severe	Group I (MIH): total removal of hypomineralised tissue. Group II (MIH): partial removal of hypomineralised tissue. Group III (MIH): partial removal of hypomineralised tissue + deproteinization. Group IV (no MIH): control	Group I had a retention rate of 93.7%, Group II had a retention rate of 80.7%, Group III had a retention rate of 93.5%, and Group IV had a retention rate of 100%. Deproteinization of hypomineralized enamel was observed to improve CRR retention rates
Muniz et al. (2019) [74]	RCT	66	8.89 years old	31 males, 35 females	n.d.	The patients were randomly allocated to three treatment groups: (A) laser (LLLT) group: the participants in this group underwent two sessions of low-level laser therapy (LLLT) with an infrared laser diode (wavelength: 808 nm) applied to the teeth. (B) fluoride varnish (FV) group: this group received four applications of fluoride varnish (Duraphat^®^, 22,600 ppm F) at one-week intervals. (C) Laser + fluoride varnish (L + FV) group: this group underwent two sessions of LLLT and four applications of fluoride varnish, following the same procedures as the L and FV groups, respectively	Fluoride varnish and the combination of treatments (L + FV) had a greater desensitizing effect on teeth with MIH. Laser therapy (LLLT) demonstrated an immediate desensitizing effect, while fluoride varnish had a late-onset effect
Gatón-Hernandez et al. (2020) [57]	RCT	326 patients	6–8 years old	172 males, 154 females	The severity of MIH was categorized as severe (with enamel breakdown and cavities) and was associated with sensitive teeth	The treatment involved minimally invasive procedures, including the selective removal of carious tissue, restoration with glass ionomer cement followed by resin composite, and preventive measures such as dietary counseling, oral hygiene instructions, plaque control, and topical fluoride application	The study measured clinical and radiographic outcomes, including success rates, the absence of sensitivity, integrity of restoration margins, absence of pathological radiographic alterations, and physiological apexogenesis
Linner et al. (2020) [75]	RCT	52	11.2 years old	26 males, 26 females	Different degrees	The study examined various treatments, including GIC restorations, non-invasive composite restorations, conventional composite restorations, and CAD/CAM ceramic restorations.	Success rate after 36 months: GIC restorations, 7.0%; non-invasive adhesive composite restorations, 29.9%; conventional composite restorations, 76.2%; CAD/CAM ceramic restorations, 100.0%
Murri Dello Diago and Patricia Gatón-Hernandez et al. (2021) [76]	RCT	67	6 to 14 years old	n.d.	enamel defects of molars due to MIH, according to diagnostic criteria proposed by the European Academy of Pediatric Dentistry (EAPD) in 2010	Surface resin infiltration using ICON	Improvement in sensitivity, plaque accumulation, and gingival inflammation after treatment
Nogueira et al. (2021) [77]	RCT	51	8.1 years old	25 males, 26 females	Mild	FV (fluoride varnish): four applications with a one-week interval between applications. FV + etch (fluoride varnish + phosphoric acid pre-treatment): four applications with a one-week interval between applications. RI (resin infiltration): one application	After the 18-month assessments, the frequency of failure was 17.9% for FV, 17.3% for FV + etch, and 6.10% for RI. Failures mainly occurred in molars. Carious lesion development (CL) was only associated with PEB for molars treated with FV (2 teeth; 4.4%) or FV + etch (1 tooth, 2.3%). The survival of FV and FV + etch was significantly lower compared to RI at 6 months
Rolim et al. (2021) [78]	RCT	35	10 years old	19 males, 16 females	Severe	The FPMs were randomized into two groups: total-etch (TE) and self-etch (SE). They were restored with a universal adhesive and bulk-fill resin composites	The study evaluated the survival rates of the restorations according to modified USPHS criteria. Dental anxiety was assessed using the Venham picture test, and dental pain was evaluated using the Faces pain scale—revised before treatment and at 1, 6, and 12 months post-treatment. Both restorative protocols had similar longevity and reduced self-reported pain and anxiety levels
Sobral et al. (2021) [79]	RCT	140	26.5 years old	n.d.	n.d.	The text describes four treatment groups: a control group (placebo), a group using PermaSeal, a group receiving low-level laser therapy (LLL), and a group combining LLL with PermaSeal.	The primary outcome is described as the change in pain/sensitivity assessed through a visual analog scale (VAS) at different time points (1 week, 1 month, 3 months, and 6 months after treatment)
Al-Batayneh et al. (2022) [80]	RCT	50	11 years old	26 females, 24 males	Moderate and severe	The study evaluated the success of different vital pulp treatment (VPT) procedures, including indirect pulp treatment (IPT), partial pulpotomy (PP), and cervical pulpotomy (CP)	PT: 96% success rate after 24 months. PP: 90% success rate after 24 months. CP: 82% success rate after 24 months. There were no significant differences in success rates between these treatment groups
Ballikaya et al. (2022) [81]	RCT	48	8.8 years old	18 females, 30 males	Mild and moderate	The study compared two treatments for MIH-affected molars: silver diamine fluoride (SDF) application alone and silver-modified atraumatic restorative treatment (SMART). SDF was applied to one group of molars, while SMART sealants were applied to another group. SMART involved treating the carious lesion with SDF and then sealing or restoring the tooth with a glass ionomer cement	SDF and SMART sealants showed favorable short-term prevention against dental caries, while providing effective desensitization in hypomineralized molars. Marginal discoloration was noted as the most common side effect of SMART sealants due to prior SDF application. The study suggested that both SDF and SMART sealants are effective in the short term for managing enamel caries and reducing hypersensitivity in hypomineralized molars
Butera et al. (2022) [82]	RCT	25	8.6 years old	6 males and 19 females.	Mild	The patients were provided with a domiciliary hydroxyapatite-based paste to be applied on MIH-affected teeth.	The biomimetic zinc-hydroxyapatite-based paste showed a desensitizing effect when used to treat MIH. The paste appeared to reduce dental sensitivity and led to a slight reduction in MIH-TNI scores in teeth with mild MIH defects
Luppieri et al. (2022) [83]	RCT	15	n.d.	n.d.	Severe	The study evaluated the effects of resin infiltration (RI) technique and the changes in PFM surfaces using profilometric and scanning electron microscope (SEM) analysis at different times: baseline (T0), right after treatment (T1), one-week follow up (T2), and one-, two-, and three-month follow up (T3, T4, and T5)	The resin infiltration resulted in smoother surfaces at T1, but there was a progressive increase in superficial roughness over time. Hypersensitivity improved at T1 and remained stable over time in severe MIH cases. Resin infiltration appears promising
Mendonça et al. (2022) [84]	RCT	60	8 years old	n.d.	Mild and moderate	Three treatment groups: control group (sodium fluoride varnish), Experimental Group I (4% titanium tetrafluoride varnish), and Experimental Group II (surface pre-reacted glass ionomer filler-containing coating resin)	The primary outcome is the level of sensitivity measured at different time points using the Wong–Baker FACES pain rating scale, Schiff Cold Air Sensitivity Scale, and FLACC (Face, Legs, Activity, Cry, Consolability) scale. Secondary outcomes include parental satisfaction and self-reported discomfort by the child
Olgen et al. (2022) [85]	RCT	49	6–9 years old	23 females, 26 males	Mild and moderate	Fluoride varnish group: Applied 5% sodium fluoride varnish. CPP-ACP group CPP-ACFP group Control group: Given oral hygiene training.	Mild and Moderate MIH: all remineralization agents increased remineralization rates evaluated by DIAGNOdent Severe MIH: unsuccessful
Özgür et al. (2022) [86]	RCT	39	8.6 ± 1.4 years old	13 females, 26 males	Mild and moderate	The treatment involved the application of two types of sealants: Group 1: resin sealant Group 2: giomer sealant	Over a 12-month trial period, conventional resin-based sealants outperformed giomer sealants that were applied with a self-etch primer. The color or extent of the MIH lesion had no influence on the survival of sealants
Sezer et al. (2022) [3]	RCT	53	8–12 years old	28 females, 25 males	Mild, moderate	CaGP: (R.O.C.S.^®^ Medical Minerals Gel, Unicosmetic OU, Tallin, Estonia, includes CaGP, magnesium, and xylitol, R.O.C.S. Trading GmbH, Munich, Germany)—27 children CPP-ACFP: (10% CPP-ACP plus 0.2% NaF; GC MI Paste PlusTM, GC Europe)—16 children Control (routine dental home care with Colgate Toothpaste including 1450 ppm F, Colgate Oral Pharmaceuticals, New York, NY)—10 children	Remineralization was evaluated vis LF (DIAGNOdent™ Pen, KaVo Dental, Biberach an der Riss, Germany). Significant changes in LF scores in time or between the groups (*p* < 0.001). Remineralization was achieved over a three-month period in both experimental groups (*p* < 0.05). In the control group, remineralization was observed over time in lesions that scored less than 20 (*p* < 0.001), although no significant changes in lesions that scored greater than 20 over time (*p* > 0.05)
Vicioni-Marques et al. (2022) [87]	RCT	23	8.4 years old	15 males, 8 females	Moderate	The children received either ibuprofen (analgesic) or a placebo before undergoing restorative dental treatment	The study evaluated the effectiveness of preventive analgesia in alleviating hypersensitivity during and after restorative treatment of permanent molars affected by MIH with post-eruptive enamel breakdown (PEB). The outcomes included the assessment of hypersensitivity at different time points after analgesic or placebo administration
Fossati et al. (2023) [88]	RCT	50	9 years old	n.d.	Grade 3 and grade 4 according to the MIH Treatment Need Index.	Two treatment groups are described: Group 1, which includes fluoride toothpaste, glass ionomer sealant, and simulated low-level laser (LLL), and Group 2, which includes fluoride toothpaste, glass ionomer sealant, and active LLL treatment	The outcomes to be assessed included MIH registration, Simplified Oral Hygiene Index (OHI), Cold Air Sensitivity Schiff Scale (SCASS), Visual Analog Scale (VAS) for sensitivity, and sealant retention. These assessments were carried out before the procedure, immediately after the procedure, at 48 h post procedure, and one month post procedure
Hakmi et al. (2023) [89]	Clinical trial	20 (40 teeth)	8.3 years old	11 males, 9 females	Severe	The study compared two types of restorative treatments: direct composite resin restoration (DCRR) Indirect Composite Resin Restoration (ICRR)	ICRR demonstrated advantages in terms of child satisfaction due to shorter treatment sessions. The survival rate after 12 months was 85% for DCRR and 90% for ICRR
Kaya et al. (2023) [90]	RCT	48	8.8 years old	18 females, 30 males	Mild and moderate	The study compared two treatments for MIH-affected molars: silver diamine fluoride (SDF) application alone and silver-modified atraumatic restorative treatment (SMART). SDF was applied to one group of molars, while SMART sealants were applied to another group. SMART involved treating the carious lesion with SDF and then sealing or restoring the tooth with a glass ionomer cement	SDF and SMART sealants showed favorable short-term prevention against dental caries while providing effective desensitization in hypomineralized molars. Marginal discoloration was noted as the most common side effect of SMART sealants due to prior SDF application. The study suggested that both SDF and SMART sealants are effective in the short term for managing enamel caries and reducing hypersensitivity in hypomineralized molars

## Data Availability

Not applicable.

## Abbreviations

CaGP	calcium glycerophosphate
CPP-ACFP	casein phosphopeptide amorphous calcium fluoride phosphate
CPP-ACP	casein phosphopeptide-amorphous calcium phosphate
CS	case series
DCRR	direct composite resin restoration
DED	developmental enamel defects
EDI	enamel defects index
FPM	first permanent molars
FV	fluoride varnish
FV + etch	fluoride varnish with phosphoric acid pre-treatment
GH	glass hybrid
GIC	glass ionomer cement
ICDAS	International Caries Detection and Assessment System
ICRR	Indirect Composite Resin Restoration
LF	laser fluorescence
LLLT	low-level laser therapy
MIH	molar incisor hypomineralization
PEB	post-eruptive breakdown
PFMs	permanent first molars
QLF	quantitative light-induced fluorescence
RCCT	Randomized Controlled Clinical Trial
RCT	randomized control trial
RI	resin infiltration
SAI	Schiff Air Index
SDF	silver diamine fluoride
SEA	selfetching adhesive
SFRC	short-fiber-reinforced composite
SMART	silver-modified atraumatic restorative treatment
TEA	total-etching adhesive
TNI	Treatment Need Index
